# Mental Practice Ability Among Stroke Survivors: Investigation of Gender and Age

**DOI:** 10.3389/fpsyg.2019.01568

**Published:** 2019-07-10

**Authors:** Vera Storm, Till Utesch

**Affiliations:** Department of Sport and Exercise Psychology, Institute of Sport and Exercise Sciences, University of Münster, Münster, Germany

**Keywords:** stroke, mental practice ability, motor imagery, gender differences, age differences

## Abstract

**Background:** Mental practice refers to the imaginary representation of a motor action. Mental practice interventions are frequently used among stroke survivors to improve motor function. Individual characteristics that may determine whether a person is able to mentally perform a specific movement have been mainly spared in research.

**Aims:** The aim of the present study is to examine whether gender and age are related to mental practice ability.

**Methods:** The study has a cross-sectional design. Data collection was done *via* self-report questionnaires on mental practice ability, sociodemographic information, and perceived stroke impact. Data analysis was conducted in R using descriptive statistics and regression analysis. *N* = 44 stroke survivors (*M* = 65.8 years, SD = 11.4, range 48–88), *n* = 19 of which were female were recruited in two German neurologic rehabilitation facilities.

**Results:** Age (*β* = −0.13, *p* = 0.057) and gender (*β* = 0.17, *p* = 0.260) were not associated with mental practice ability, when controlling for time since stroke and perceived stroke impact (Stroke Impact Scale). Perceived stroke impact was significantly related to mental practice ability (*β* = 0.44, *p* = 0.004). Those who reported less stroke impact showed better mental practice ability.

**Conclusion:** Mental practice ability may be preserved in stroke patients, irrespective of age and gender. We report cross-sectional data on mental practice ability in this study, thus the direction of the relationship between mental practice ability and perceived stroke impact is of interest. Future studies should aim at using a longitudinal design and bigger sample sizes.

## Introduction

Stroke is one of the most common diseases among adults worldwide and is frequently connected with cognitive and physical disabilities (Thrift et al., 2017). Due to improved medical care and living circumstances, mortality rates have decreased during the last decades in industrialized countries like Germany (Schmidt et al., 2014). However, motor deficits, memory deficits (Cumming et al., 2013), speech problems (Pedersen et al., 2004), and depression (Hackett and Pickles, 2014) are still common among stroke survivors.

The majority of psychological interventions and rehabilitation programs during the neurological recovery process focus on coping (Eldred and Sykes, 2008; Hackett et al., 2008) and treatment of cognitive disabilities (Gittler and Davis, 2018) instead of motor functions. Meanwhile, other cognitive-oriented therapies such as mental practice are discussed. Mental practice (MP) refers to the imaginary representation of a motor action or skill (Braun et al., 2008) in the sense of mentally performing an action instead of physically performing it. Functional imaging has shown that MP produces similar cortical activation patterns to those of actual movement (Liu et al., 2014). MP provides an attractive alternative to other rehabilitative approaches because it “does not require physical rehearsal, can be performed without direct supervision, and requires minimal expense and equipment, facilitating ease of use” (Page and Peters, 2014, p. 3454).

Current meta-analyses show that MP interventions seem to be effective in improving upper and lower extremity function among stroke patients (Zimmermann-Schlatter et al., 2008; Kho et al., 2014; Li et al., 2017). The mechanisms that are hypothesized to be responsible are use-dependent cortical organizations and overcoming nonuse of the affected limb (Page and Peters, 2014).

Based on the qualitative descriptions in a meta-analysis by Braun et al. (2013), MP interventions seem to have potential irrespective of gender, in all ages of participants and phases of stroke recovery but systematic research is missing. For the development and tailoring of such interventions to a person’s special needs, it is important to characterize which persons are able to mentally perform a certain movement.

MP ability has been largely documented in young and healthy adults. However, due to the fact that the use of mental practice in stroke rehabilitation involves mainly aging populations, it is important to investigate how MP ability is related to age. Because MP involves the generation and maintenance of motor representations in working memory, one could assume that older participants score lower on MP tests due to decreased working memory capacity. Indeed, Malouin et al. (2004) showed that performance of MP is related to working memory capacity. However, age did not predict MP ability in studies by Malouin et al. (2007, 2010) and Butler et al. (2012), indicating that MP ability in older adults can be as good as in young adults.

Research on gender differences is also heterogenous and mainly applied to healthy populations. In a study by Isaac and Marks (1994), healthy women reported better and more vivid MP ability than healthy men who match the results of an earlier meta-analysis by Richardson (1995). However, Subirats et al. (2018) did not find any gender differences.

In order to design effective tailored intervention programs for patients recovering from stroke and close the research gaps on age and gender differences in MP ability, it is important to obtain detailed information among stroke patients. Consequently, we aim to fill the literature gap by evaluating MP ability and considering the impact of both age and gender.

## Materials and Methods

### Study Design

The present study has a cross-sectional study design. The study has been pre-registered within the open science framework (OSF) on https://osf.io/w7jex/. The study protocol has been approved by the ethics committee of the University of Münster (Department of Sport Sciences and Psychology) and is in accordance with ethical standards on human experimentation and with the Helsinki Declaration of 1975, as revised in 1983.

### Procedure and Participants

The recruitment took place between March and May 2018 in two German neurologic rehabilitation facilities. Data were obtained by trained researchers from participants who agreed by written consent. The inclusion criteria required being aged 18 years and above, being diagnosed with stroke, being able to differentiate between left and right, and being able to communicate in German. The exclusion criteria were suffering from aphasia or other severe communication disorder or visual deficits.

### Measurements

Data were obtained by self-report questionnaires in German language. Socio-demographic variables included year of birth, gender, highest educational status, marital status, and employment status.

#### Mental Practice Ability

MP ability was assessed with the visual imagery scale of the short form of the Kinesthetic and Visual Imagery Questionnaire (KVIQ) by Malouin et al. (2007). The visual imagery scale was chosen for parsimony reasons. The KVIQ is used among different clinical samples such as Parkinson’s disease (Randhawa et al., 2010) and stroke (Malouin et al., 2008). The short version includes 10 items (five movements for each scale). For each item (for example, foot tapping or thumb to finger tips), the examiner asks the participant to perform a movement from a seated position, once only. Then, the participant is requested to imagine performing the movement that he or she just executed while not actually performing the movement. After that, the participant is asked to rate the clarity of the visual image on a 5-point ordinal scale ranging from no image (1) to image as clear as seeing (5). A sum score is calculated for the scale. Cronbach’s *α* was 0.88.

#### Stroke Impact

Perceived stroke impact was assessed by the short version of the Stroke Impact Scale (SIS; MacIsaac et al., 2016). The SIS is a stroke-specific quality of life measure. For each of the eight items, the participant is asked to rate the level of difficulty of the item in the past 2 weeks using the following scale: 1 = could not do it at all, 2 = very difficult, 3 = somewhat difficult, 4 = a little difficult, and 5 = not difficult at all. A sum score is calculated for the scale. Higher values indicate better quality of life. Cronbach’s *α* was 0.76.

### Data Analysis

Data analysis was conducted in R (R Core Team, 2013) mainly with the packages psych (Revelle, 2018), sj.Plot (Lüdecke, 2017), and RSA (Schönbrodt and Humberg, 2016) using descriptive statistics and regression analysis. In the main analysis, months since stroke were included as a covariate. Open code and open data are provided in an online supplement (https://osf.io/w7jex/).

## Results

A summary of the main study variables is provided in Table 1.

**Table 1:** Summary of main study variables and intercorrelations.

	1	2	3	*M*	SD	Range
1. Mental practice ability				40.54	5.5	27–45
2. Months since stroke	−0.01			29.42	6.36	0.5–115
3. Perceived stroke impact	0.46*	−0.02		65.71	11.72	18–40
4. Age	−0.26	−0.18	−0.02	1.07	0.78	48–88

*indicates p < 0.05.

### Descriptive Results

After obtaining informed consent, *n* = 44 persons were assessed with paper-and-pencil questionnaires with mean age 65.8 years, standard deviation (SD) 11.4 years, and age range 48–88 years. About 43.2% (*n* = 19) were female. Of the participants, 78.6% (*n* = 33) were married or in a relationship, and 31.7% (*n* = 13) were employed either full time or part time. The time since stroke ranged between 1 month and 19 years. *n* = 35 of the participants reported to have an affected leg, and *n* = 35 mentioned to have an affected arm. All of the participants indicated to be right-handed. Time since stroke and perceived stroke impact were included as covariates.

### Main Results

**Figure 1:**
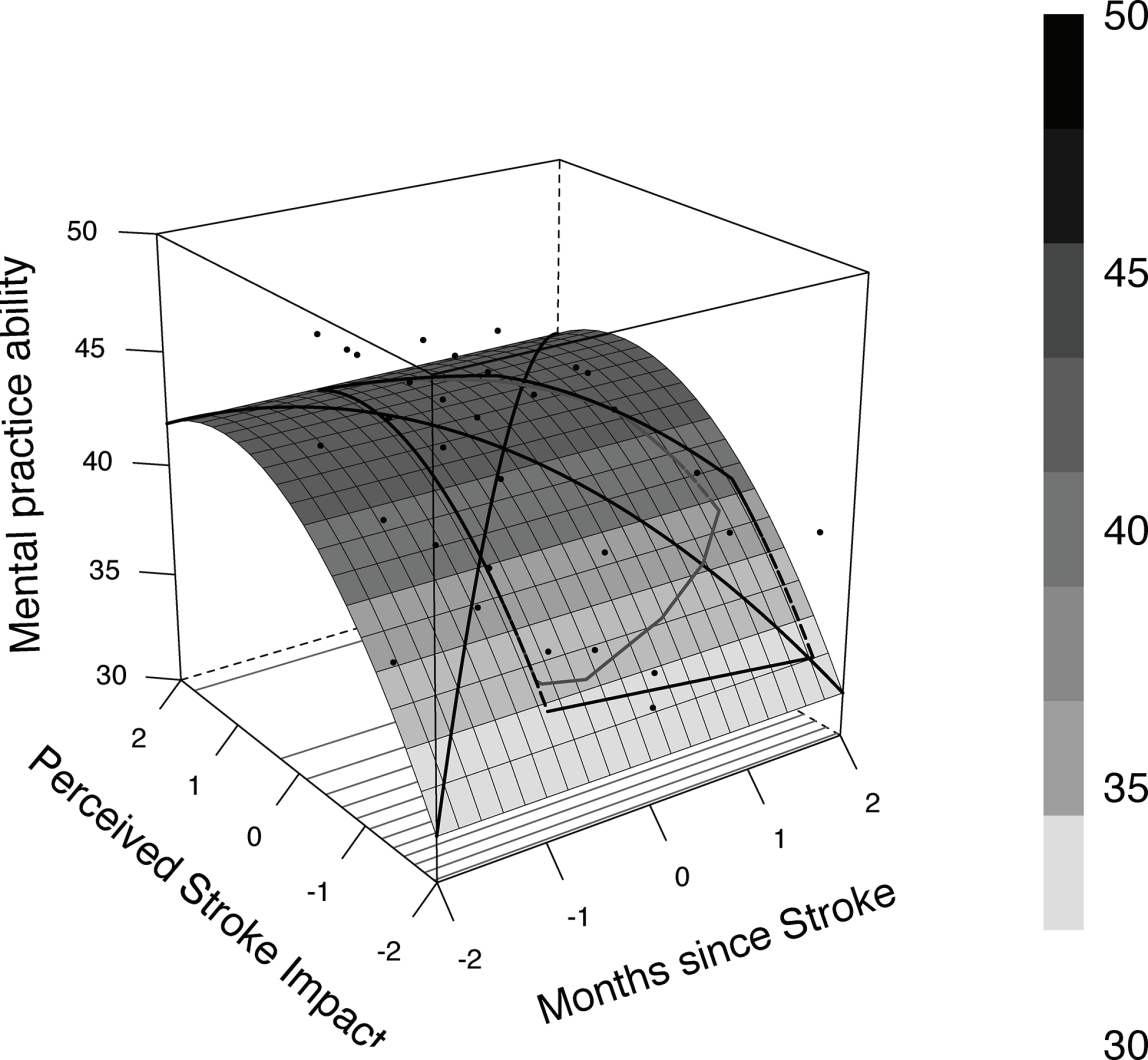
Relationship between perceived stroke impact, months since stroke, and mental practice ability.

A total of 32.1 (adjusted 24.6%) of the variance was explained by the predictors. Age (*β* = −0.13, *p* = 0.057) and gender (*β* = 0.17, *p* = 0.260) were not associated with MP ability when controlling for time since stroke and stroke impact. The detailed results of the regression analysis are portrayed in Table 2. Further exploratory analysis revealed a curvilinear relationship between MP ability and perceived stroke impact (c.f., Figure 1).

**Table 2:** Regression analysis on the dependent variable mental practice ability.

Predictors	*B*	*B* 95% CI	*β*	*t*	*p*
Age	−0.13	−0.26; 0.01	−0.28	−1.97	0.057
Gender	1.77	−1.37; 4.91	0.17	1.14	0.260
Perceived stroke impact	0.38*	0.13; 0.62	0.44	3.12	0.004
Months since stroke	−0.02	−0.97; 0.94	−0.01	−0.03	0.974
Observations	40				
*R*^2^/adjusted *R*^2^	0.32/0.25				

Computed correlation used Pearson’s method with listwise deletion. *indicates p < 0.05.

## Discussion

Given the potential of MP interventions among stroke patients, it is important to obtain detailed information about MP ability among stroke patients. Consequently, in this study, we examined whether age and gender were related to MP ability. We did not find any age-related effect regarding the ability to mentally perform a certain action. This matches the findings shown by Malouin et al. (2010) as well as Schott (2012) who also found that MP ability in older adults was as good as in young adults. For future studies, it would be interesting to investigate not only whether the level of MP remains stable with age but also whether its actual quality changes, e.g., due to an age-related decline in visuospatial and kinesthetic working memory. For instance, Subirats et al. (2018) propose that there is an age-related transfer from a visual to a kinesthetic MP ability. Therefore, full scales of MP ability are recommended (Malouin et al., 2007) as well as potential behavioral assessments.

In the current sample, sex did not significantly predict MP ability scores. Research on gender differences so far showed mixed results. While Schott (2013) reports similarities between men and women, others report that women perform better in timing (Subirats et al., 2018) or men better in visuospatial content. In summary, researchers should tailor MP interventions considering all patient characteristics when developing post-stroke treatments and not sex and age in specific.

It should be acknowledged that we report cross-sectional data in this study; thus, the directions of the relationships are of interest in future studies. In addition, researchers should aim at using a longitudinal design with bigger sample sizes and include the full KVIQ scale that was abbreviated for parsimony reasons here.

## Data Availability

The raw data supporting the conclusions of this manuscript will be made available by the authors, without undue reservation, to any qualified researcher.

## Ethics Statement

The study protocol has been approved by the ethics committee of the University of Münster (Department of Sport Sciences and Psychology) and is in accordance with ethical standards on human experimentation and with the Helsinki Declaration of 1975, as revised in 1983.

## Author Contributions

VS and TU made substantial contributions to the conception or design of the work. TU was responsible for the analysis of data for the work. VS drafted the work and together with TU revised it critically for important intellectual content. Both authors provide approval for publication of the content. Both authors agree to be accountable for all aspects of the work in ensuring that questions related to the accuracy or integrity of any part of the work are appropriately investigated and resolved.

### Conflict of Interest Statement

The authors declare that the research was conducted in the absence of any commercial or financial relationships that could be construed as a potential conflict of interest.
